# The interaction of peptide inhibitors and Aβ protein: Binding mode analysis, inhibition of the formation of Aβ aggregates, and then exert neuroprotective effects

**DOI:** 10.3389/fnagi.2023.1139418

**Published:** 2023-04-11

**Authors:** Yuchen Wu, Shuang Guo, Kunli Wang, Jingjing Kang

**Affiliations:** ^1^College of Veterinary Medicine, Henan University of Animal Husbandry and Economy, Zhengzhou, China; ^2^College of Veterinary Medicine, Henan Agricultural University, Zhengzhou, China

**Keywords:** Aβ, molecular docking, neuroprotection, neuroscience, PC12 cells +

## Abstract

**Introduction:**

The misfolding and aggregation of β-amyloid (Aβ) easily form Aβ fibers, which are continuously deposited in the brain, leading to the massive generation of amyloid plaques, severely destroying neuronal connections, and promoting Alzheimer’s disease (AD) The occurrence and development of AD is one of the pathogenesis of AD. There is an urgent need to develop inhibitors against Aβ aggregation, which is hopefully a potential way to treat AD.

**Methods:**

In this study, we first found the crystal structure of the Aβ_1–42_ receptor protein from the RCSB PDB protein structure database and used the SYBYL X2.0 software for molecular docking, and then used the Peptide Ranker, Innovagen, DPL, and ToxinPred online websites to perform peptides. Predict the activity score, toxicity and water solubility, and then calculate the affinity constant KD value of polypeptide and Aβ through Surface Plasmon Resonance (SPR) experiment. Subsequently, the CCK-8 kit method was used to determine the toxicity of different concentrations of peptides (3.125, 6.25, 12.5, 25, 50, 100, 200 μM) to PC12 cells, and then the peptides and Aβ according to different concentration ratios (1:4, 1:2, 1:1, 1:0.5, 1:0.25, 0:4), this method is also used to detect the effect of peptides on Aβ-induced neurotoxicity. The thioflavin T (ThT) fluorescence method was used to detect the effects of peptides (50 μM) on Aβ (25 μM) aggregation inhibitory effect.

**Results:**

The results showed that the CScore of YVRHLKYVRHLK peptide molecule docking was 10.0608, the predicted activity score was 0.20, and the KD value was 5.385 × 10−5. The ThT and CCK-8 kit method found that the peptide itself is less toxic to PC12 cells at a concentration of 50 μM, and it has a significant inhibitory effect on the formation of Aβ_1–42_ aggregates when incubated with Aβ_1–42_ at a ratio of 1:1 (p < 0.05) and can significantly reduce the PC12 cytotoxicity induced by Aβ_1–42_ (p < 0.05).

**Conclusion:**

In conclusion, the polypeptide YVRHLKYVRHLK designed in this study has a neuroprotective effect on PC12 cytotoxicity induced by Aβ_1–42_.

**Graphical Abstract:**
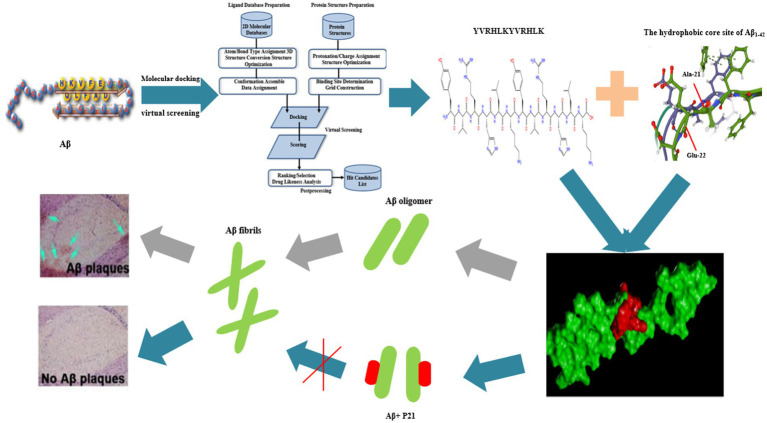

## 1. Introduction

Alzheimer’s disease (AD) is a progressive neurodegenerative disease of the central nervous system that occurs in the elderly. The main pathological feature is the formation of amyloid senile plaques, the main component of which is β-amyloid protein (Aβ), its misfolding and excessive aggregation trigger the disease process of AD, and it is one of the core factors in the occurrence and development of AD (Lin et al., 2014). Under normal circumstances, amyloid precursor protein (APP) is produced under the cleavage of α-secretase (ADAM10) and does not produce Aβ. Instead, it produces neurotrophic factor (sAPPα), which is beneficial to human nerves, which is the non-starch of APP. The amyloidogenic pathway of APP is the enzymatic reaction of β-secretase (BACE1) and γ-secretase (PS1) to produce Aβ. Studies have found that potentiating BACE1 cleavage of APP at both the Asp1 and Glu11 sites, or shifting the cleavage from the Glu11 site to the Asp1 site, can greatly increase the level of C99 and the ratio of C99/C89, increased Aβ production and facilitate neuritic plaque formation (Deng et al., 2013; Zhang et al., 2017). The production and elimination of Aβ in the human body is in a balanced state, but any mutation of presenilin-1 (PSEN), presenilin-2 (PSEN 2) and amyloid precursor protein (APP) genes will cause People suffer from sporadic AD (SAD) or late-onset AD (LOAD) (Uddin and Kabir, 2020). Aβ is composed of 42 amino acids, and α-helix, β-turn and β-sheet form its secondary structure. The β-sheet constitutes the hydrophobic carboxyl terminus, while the α-helix and β-turn constitute the hydrophilic amino terminus. Under physiological conditions, the hydrophobic carboxyl end is hidden and the hydrophilic amino end is exposed, and Aβ is soluble (Wang et al., 2004). Multiple evidence indicated that a large part of Aβ aggregation is driven by hydrophobic sequences (Hilbich et al., 1992; Jarrett et al., 1993; Soto et al., 1995).

Nowadays, the drugs that slow the progression of AD on the market mainly include acetylcholinesterase inhibitors (rivastigmine, galantamine and donepezil), N-methyl-D-aspartic acid (NMDA) antagonists (memantine), intestinal flora regulator (sodium oligomannate, GV-971), Aβ inhibitors (Aducanumab, Gantenerumab, Ban2401 and Alz-801), among which drugs such as rivastigmine, galantamine, donepezil and memantine also have potential side effects, such as Diarrhea, vomiting, nausea, and abdominal cramps (Wang et al., 2019; Behl et al., 2020; Tolar et al., 2020). Aducanumab is a recombinant antibody that uses the epitope mapping and binding kinetics of synthetic peptides to study the crystal structure of the antigen-binding region (Fab) of the aducanumab fragment that binds to Aβ_1–11_. Computer analysis showed that aducanumab has a weak binding effect to the N-terminal of Aβ, and may adapt to a variety of peptide conformations, which further supports its selectivity for Aβ aggregates (Arndt et al., 2018). In current research, inhibitors for Aβ_1–42_ protein aggregation have a certain inhibitory effect in previous studies, but the results in subsequent studies are not ideal. Therefore, there is an urgent need to develop a peptide inhibitor against Aβ aggregation. This research creatively uses computer molecular docking technology to simulate and design peptide inhibitors targeting the structural sites of Aβ_1–42_. First, predict the activity score, electrostatic charge, toxicity, and water solubility of the peptide, and then use the CCK-8 reagent. The box method was used to determine the toxicity of the polypeptide itself to PC12 cells and the effect of the polypeptide on the neurotoxicity induced by Aβ_1–42_. Then, the inhibitory effect of the peptide inhibitor on the aggregation of Aβ_1–42_ was detected by the thioflavin T (ThT) staining method. One step is to develop functional foods that can prevent AD and drugs to treat AD to lay a theoretical foundation.

## 2. Materials and methods

### 2.1. Experimental materials and reagents

Hexafluoroisopropanol (HFIP) and ThT were purchased from Solarbio. The CM5 chip was purchased from GE in the United States. Human-derived Aβ_1–42_ standard (95%), P9(Ac-YVRHHHYVRHHH-NH2), P11(Ac-YVRHSVYVRHSV-NH2), P12(Ac-YVRHDLYVRHDL-NH2), P21(Ac-YVRHLKYVRHLK- NH2) was purchased from GL Biochem Ltd. (Shanghai). Cell Counting Kit-8 was purchased from Biosharp Biotechnology (Beijing). The remaining reagents can be purchased from suppliers and can be used without purification.

### 2.2. Preparation of Aβ_1–42_ oligomer solution and preparation of peptide samples

Rat adrenal pheochromocytoma (PC12) cells were donated by Zhengzhou University School of Basic Medicine (#CRL-1721; RRID: CVCL-0481) in DMEM containing 10% fetal bovine serum, 1% penicillin and 1% streptomycin, culture in a cell incubator with 5% CO_2_ and 37°C, and passage once every 3 days.

Take the Aβ_1–42_ monomer out of the refrigerator at −20°C and place it on ice. Add 0.11 mL of HFIP pre-cooled on ice in advance for every 0.5 mg of Aβ_1–42_ monomer and operate on ice throughout the process. Let stand at room temperature for 1 h to fully dissolve Aβ_1–42_ and dispense 100 μL per tube. Make HFIP volatilize completely in a fume hood, and freeze at −80°C. Then add DMSO to the centrifuge tube to fully dissolve it to obtain a 2 mmol/LAβ_1–42_-DMSO solution. Place the centrifuge tube in an ultrasonic bath for 10 min to fully dissolve the peptide membrane. Add phenol red-free DMEM medium to each tube to dilute to the final concentration required for the experiment (Messa et al., 2014).

First dissolve the peptides in ultrapure water to make a 10 mg/ml stock solution and dispense them into 1.5 mL EP tubes. Dilute the stock solution to the corresponding concentration as needed during use to avoid repeated freezing and thawing.

### 2.3. Molecular docking and virtual peptide library screening

Search the known crystal structure of Aβ_1–42_ active ingredient from Protein Data Bank, PDB code is 6SH3, as a molecular pair acceptor model. Using FlexX, a semi-flexible, fast and accurate ligand docking method based on incremental construction, the interaction between peptides and Aβ_1–42_ protein is reflected through FlexX/SYBYL X2.0 software, and Surflex-Dock (SFXC) is selected as the docking mode to generate protomol Related documents, the docking results are expressed relative to the best docking mode. In order to confirm the correctness and reliability of the docking model, before the target small molecule docking, according to the experimental working parameters, the original ligand molecule in the crystal structure was docked to the processed receptor structure active pocket for docking. The total score (total score) is the evaluation criterion. The total score of docking scoring is also called total surflexdock score in English. It divides the binding energy into multiple energy items, such as van der waals force, hydrogen bond, ionic bond, and hydrophobic interaction. Each energy item is multiplied by a coefficient, and the sum is finally calculated to give the final Scoring. The computer virtually screens out the peptides that bind to the Aβ_1–42_ protein.

### 2.4. Peptide’s electrostatic charge, biological activity, water solubility and toxicity prediction

Use the online tool Peptide Ranker^1^ program to predict the biological activity of the peptide inhibitors obtained in the previous stage. Use the online tool DPL^2^ Evaluate the water solubility of the designed peptide. When the net charge value is ≥2 or ≤−2, the designed peptide has good water solubility. Use the online website ToxinPred^3^ to predict the toxicity of peptides. ToxinPred can be used to predict the toxicity of peptides, in addition to designing the least toxic peptides and discovering toxic regions in proteins.

### 2.5. SPR determines the affinity constant of peptide and Aβ_1–42_

Surface Plasmon Resonance (SPR) is a sensitive surface analysis technique that detects changes in dielectric constant caused by molecules adsorbed on heavy metal films. In this study, the 1-(3-Dimethylaminopropyl)-3-ethylcarbodiimide hydrochloride (EDC) and N-Hydroxy succinimide (NHS) in the coupling kit were, respectively, dissolved in 1 mL of activation buffer, divided into 10 parts, 100 μL/part, and stored at −20°C for later use. Before the experiment, first install the CM5 chip in the machine and wash it with HBS-EP buffer 3 times. After screening the appropriate buffer pH, couple the Aβ_1–42_ protein to the chip, and then dissolve the peptide solution in HBS-EP, dilute to different concentrations, and set a zero concentration and a minimum concentration repeated sample for affinity analysis Perform system maintenance until all samples are measured. After the experiment is over, use the Evaluation software to analyze the results, and use Flow Cell2 to subtract the background signal of channel 1 as the experimental result. The 1:1 binding method is used for fitting calculation and analysis of affinity parameters: binding rate constant ka (1/Ms), dissociation rate constant kd (1/s) and equilibrium dissociation constant K_D_ (M), K_D_ = kd/ ka, the smaller the K_D_ value, the stronger the affinity.

### 2.6. ThT fluorescence detection of the inhibitory effect of peptides on the aggregation of Aβ_1–42_

ThT is a chemical fluorescent dye. In recent years, studies have found that ThT can specifically bind to Aβ to enhance its fluorescence. Based on this fluorescence phenomenon, ThT is used as a kind of fluorescent indicator to detect the degree of aggregation of Aβ. In this experiment, the ThT method was used to determine the inhibitory effect of the polypeptide on the aggregation of Aβ_1–42_.

In this experiment, Aβ_1–42_ was dissolved in a small amount of DMSO and then diluted with PBS to a mother liquor of 100 μM. The peptide inhibitor was added and diluted with PBS so that the final concentration of Aβ_1–42_ was 25 μM and the final concentration of peptide inhibitor was 50 μM. The samples containing only Aβ_1–42_ and Aβ_1–42_ solution containing peptide inhibitors were incubated separately and together at 37°C for 24 h. Take about 10 μL of the sample, dilute it 20 times with ThT solution, and read the fluorescence intensity value with a microplate reader, 440 nm is the excitation wavelength, and 480 nm is the emission wavelength, and the bandwidth is 5 nm. The fluorescence intensity of the sample containing only Aβ_1–42_ was set to 100%, and the fluorescence intensity of the PBS without Aβ_1–42_ and inhibitor was set to background subtraction for normalization. The sample was repeated five times and the average value was taken.

### 2.7. Verification of peptide toxicity

#### 2.7.1. Cell count

Pipette the cultured cells in a cell culture flask repeatedly to make them evenly distributed. Use a pipete to pipete 10 μL of cell suspension and transfer to a cell counting plate for counting. Count under a microscope to calculate the number of living cells distributed in a large square composed of 16 squares on the cytometer. Cell density (pcs / mL) = (sum of cell count/4) × 10^4^ Calculate the cell concentration, repeat counting 2–3 times for each sample (the value should not be too different each time, otherwise the operation should be repeated). If there are less than 5 cells in each large square, you need to re-centrifuge to adjust the cell concentration.

#### 2.7.2. Toxicity of peptides to PC12 cells

Use CCK-8 kit to detect cell viability, reflecting the cytotoxicity of peptide inhibitors themselves. The counted PC12 cells were plated at 100 μL/well (8 × 10^4^ cells/well). After put the 96-well plate at 37°C for 12 h, discard the medium, add 100 μL of medium containing different concentrations of peptide inhibitors to each well, and continue to culture 24 h. Add 10 μL of CCK-8 solution to each well (be careful not to generate bubbles in the well, they will affect the reading of the OD (optical density) value), and continue to incubate for 2 h. The wells with DMEM medium solution containing no peptide inhibitors but containing cells were used as the control group. The wells with 100 μL DMEM medium and 10 μL CCK-8 solution but without cells were used as the blank control, and the absorbance was measured at 450 nm.


$$\mathrm{Cell Viability}\;\left(\%\right)=\frac{\mathrm{OD}\;\mathrm{administration group}-\mathrm{OD}\;\mathrm{control group}}{\mathrm{OD}\;\mathrm{control group}-\mathrm{OD}\;\mathrm{blank group}}\times 100\%$$

#### 2.7.3. Effect of peptide inhibitors on PC12 cytotoxicity induced by Aβ_1–42_ aggregation

The cell viability of each group was detected by the CCK-8 kit method, and the effect of peptide inhibitors on the PC12 cytotoxicity caused by the accumulation of Aβ_1–42_. After incubating the sample containing only Aβ_1–42_ and the peptide inhibitor co-incubated with Aβ_1–42_ for 72 h (aging), spread it in a 96-well plate, and after incubating in a 96-well plate for 12 h, discard the medium. Add 10 μL of CCK-8 solution to each well and continue to incubate for 2 h. Add 10 μL of CCK-8 solution to each well and continue to incubate for 2 h. The wells with normal DMEM medium and cell solution were used as the control group. The wells with 100 μL DMEM medium and 10 μL CCK-8 solution but no cells were used as the blank control, and the absorbance was measured at 450 nm.

### 2.8. Data analysis

All experimental data are expressed as Mean ± SEM, and SPSS16.0 software is used for statistical analysis. *p* < 0.05 indicates that the difference is statistically significant.

## 3. Results

### 3.1. Evaluation of molecular docking and virtual screening results

The molecular docking scoring results include Cscore, Crash and Polar results. The Cscore value indicates the degree of binding between the peptide ligand and the receptor protein. The better the spatial complementarity and energy matching between the two, the higher the Cscore value. The results of molecular docking in this study are shown in Table 1.

**Table 1:** The sequence and Cscore value of five peptides obtained by FlexX/SYBYL virtual screening.

Peptide name	Amino acid sequence	Cscore
P9	YVRHHHYVRHHH	10.7333
P11	YVRHSVYVRHSV	10.5312
P12	YVRHDLYVRHDL	10.4480
P21	YVRHLKYVRHLK	10.0608

### 3.2. Peptide’s electrostatic charge, biological activity, water solubility and toxicity prediction results

The biological activity, water solubility and toxicity of the four peptides obtained through virtual screening of molecular docking were predicted. The prediction results are shown in Table 2. The prediction scores of the four peptides (P9, P11, P12, and P21) in this study are 0.15, 0.07, 0.20, and 0.20. The activity scores of P12 and P12 are higher. In terms of water solubility, P9 and P11 have poor water solubility, while P12 and P21 have better water solubility. The toxicity of the polypeptide is predicted by ToxinPred, and the results show that none of the four polypeptides is toxic.

**Table 2:** Prediction of peptide biological activity, water solubility and toxicity.

Peptide name	Net charge	Activity score	Toxicity	Water soluble
P9	+4	0.15	Non-Toxin	Poor water solubility
P11	+2	0.07	Non-Toxin	Poor water solubility
P12	+1	0.20	Non-Toxin	Good water solubility
P21	+3	0.20	Non-Toxin	Good water solubility

### 3.3. Affinity determination result of peptide and Aβ_1–42_

**Figure 1:**
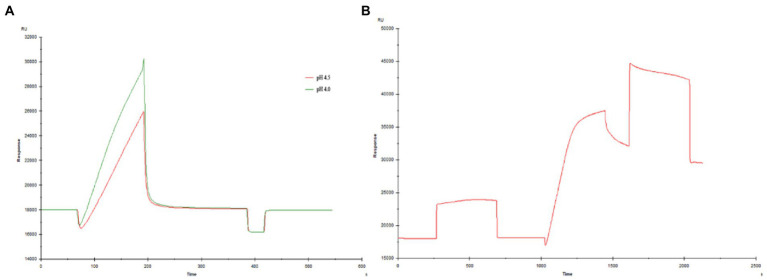
Coupling of Aβ_1–42_ and CM-5 chip **(A)** Exploration of pH conditions **(B)** Coupling result.

**Figure 2:**
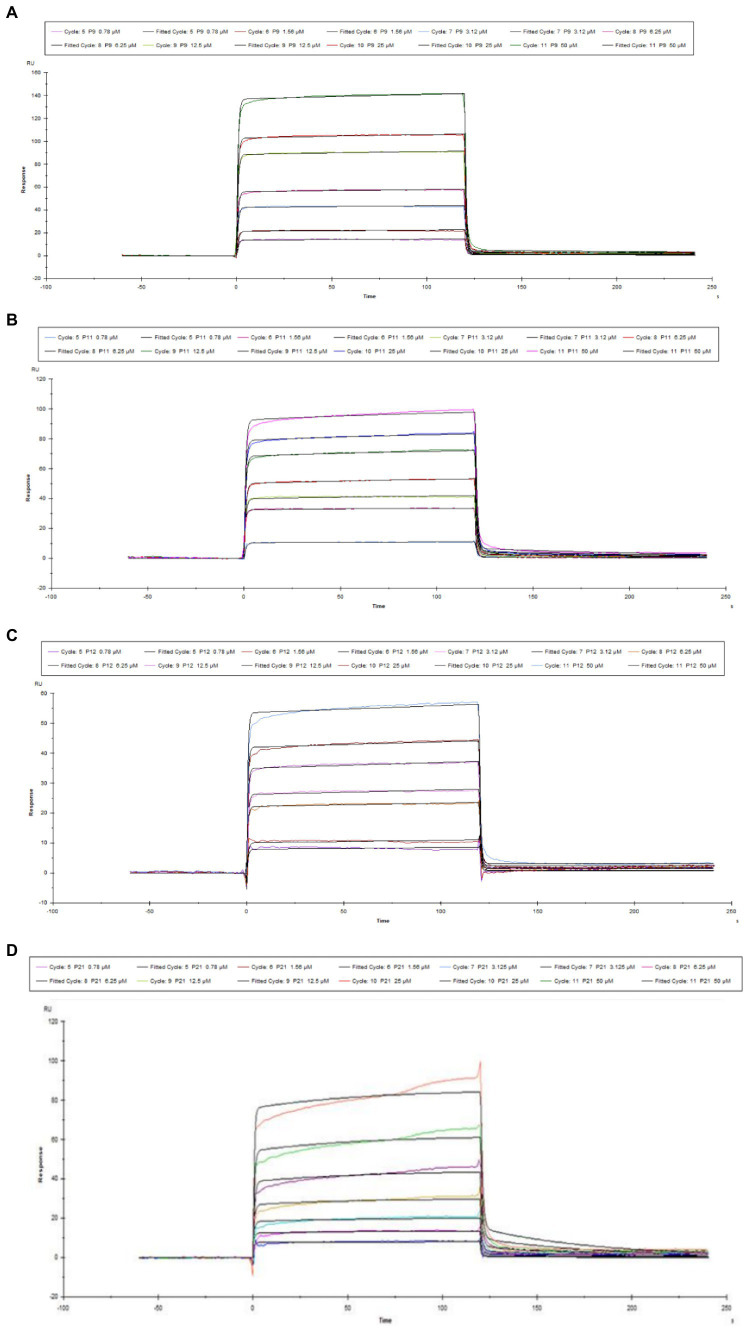
Kinetic curve diagram of the interaction between polypeptide and Aβ_1–42_. **(A)** P9 **(B)** P11 **(C)** P12 **(D)** P21.

Figure 1A shows the pH value of the sodium acetate solution coupled with the chip (for pre-enrichment of the chip). The optimal pH value was pH 4.5 in the SPR experiment. Figure 1B is the coupling result of Aβ_1–42_ and CM-5 chip. Figure 2 shows the SPR results of the interaction between peptides (P9, P11, P12, P21) and Aβ_1–42_. It can be seen from the kinetic curve in Figure 2 that the binding characteristics of different peptides to Aβ_1–42_ protein are also different. In the binding phase of polypeptide and Aβ_1–42_ protein, the faster the curve rises, the stronger the binding ability. During the dissociation phase, the faster the kinetic curve drops, the faster they dissociate. The kinetic curve of P12 polypeptide rises rapidly at the beginning of binding and reaches saturation in a short time. During the dissociation period, the curve drops rapidly and the dissociation is completed. P21 polypeptide dissociates relatively quickly, but the binding phase also rises rapidly at the beginning, but slowly reaches saturation in the following time, and the binding rate is faster. During the binding phase of P11 and P9 polypeptides, the curve rises steadily, and it takes a long time to reach saturation, and it is relatively slow in the dissociation phase. The calculation results after data fitting show that the K_D_ values of P9, P11, P12, P21 and Aβ_1–42_ are 1.401 × 10^−5^, 2.051 × 10^−5^, 3.266 × 10^−7^, 5.385 × 10^−5^ (Table 3). Since the smaller the K_D_ value, the stronger the binding ability, so the binding ability of P12 and Aβ_1–42_ is the strongest, followed by P21 and P11, and the binding ability of P9 and Aβ_1–42_ is the weakest.

**Table 3:** Kinetic parameters of the interaction between peptides and Aβ_1–42_.

Peptide name	P9	P11	P12	P21
K_D_ (M)	1.401 × 10^−5^	2.051 × 10^−5^	3.266 × 10^−7^	5.385 × 10^−5^

### 3.4. Verification of the toxicity of peptides

**Figure 3:**
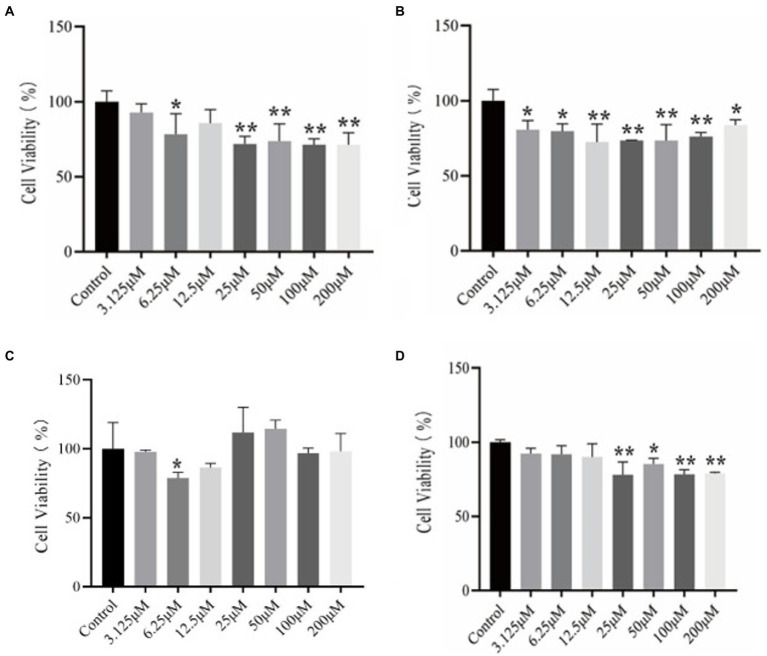
CCK-8 kit method to determine the results of different concentrations of peptides on the viability of PC12 cells. **(A)** P9 **(B)** P11 **(C)** P12 **(D)** P21.

Although online tools predict the toxicity of peptide inhibitors, in order to avoid the inaccuracy of online tools prediction, further experimental verification of the results is needed. Figure 3 is a graph showing the results of using the CCK-8 method to determine the viability of the polypeptide on PC12 cells, which reflects the toxicity of the polypeptide itself.

It can be seen from Figure 3A that the cell viability of P9 at a lower concentration (6.25 μM) began to decrease significantly (*p* < 0.05), and the cell viability of 25, 50, 100, and 200 μM also decreased significantly, with extremely significant differences (*p* < 0.01). In Figure 3B, cell viability began to decrease significantly at low concentration (3.125 μM) of P11 (*p* < 0.05), showing that this concentration can make cells significantly toxic (*p* < 0.05). Other concentrations also have significant effects on cell viability. Influence (*p* < 0.05). It can be seen from Figure 3C that P12 has significant effect on the viability of PC12 cells at 6.25 μM (*p* < 0.05). But there was no significant difference at other concentrations. In Figure 3D, the cell viability of P21 was significantly reduced at 25 μM (*p* < 0.05), and the cytotoxic peptide concentration produced at this time was higher than that of other peptides, and the cytotoxicity of P21 itself was lower.

### 3.5. The inhibitory effect of peptides on the aggregation of Aβ_1–42_ protein

**Figure 4:**
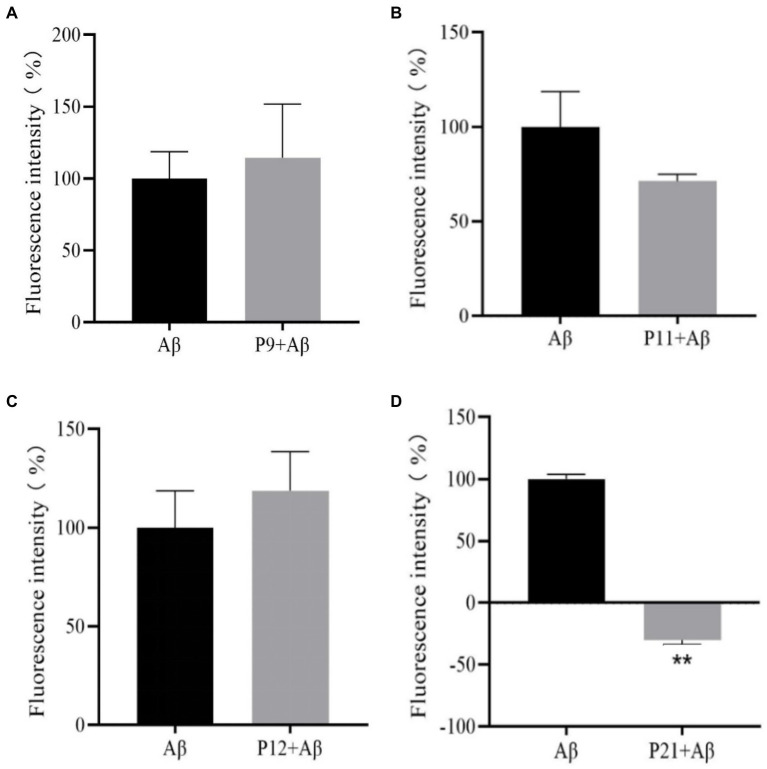
The result graph of the aggregation fluorescence intensity of peptides P9, P11, P12, P21 on Aβ_1–42_. **(A)** P9 **(B)** P11 **(C)** P12 **(D)** P21.

The previous research results showed that when the peptide concentration is 50 μM, the peptides in this experiment are almost non-toxic, and the optimal induction concentration of Aβ_1–42_ through preliminary experiments is 25 μM, so the ThT experiment is at the peptide concentration: Aβ_1–42_ concentration = 2:1. After co-culturing the peptide with Aβ_1–42_ for 24 h, the fluorescence intensity was read. The fluorescence intensity of the Aβ positive control group containing only 25 μM of Aβ_1–42_ without peptides was set to 100% for normalization. The results are shown in Figure 4.

It can be seen from Figure 4 that compared with the Aβ positive control group, the fluorescence intensity of P9 and P12 polypeptides has increased, but the difference is not statistically significant (*p* > 0.05), indicating that P9 and P12 have no inhibitory effect on Aβ aggregation. The fluorescence intensity of P11 polypeptide decreased, but the difference was not statistically significant (*p* > 0.05), indicating that P11 has a certain inhibitory ability on Aβ aggregation. The co-incubation of P21 and Aβ_1–42_ protein has a strong inhibitory ability on Aβ aggregation. P21 makes the fluorescence intensity of Aβ_1–42_ aggregation significantly lower than that of the Aβ group (*p* < 0.05), which can achieve 100% inhibition.

### 3.6. Effect of peptides on PC12 cytotoxicity induced by Aβ_1–42_ aggregation

**Figure 5:**
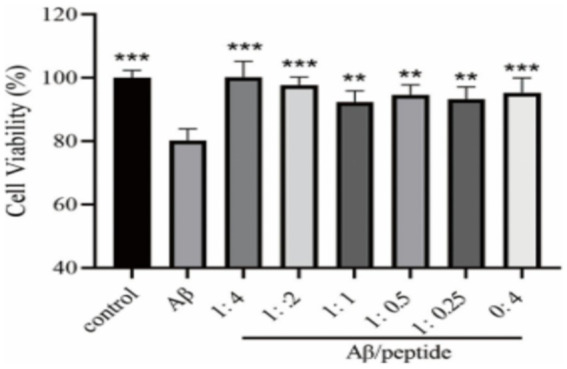
The evaluation result of P21 on the cytotoxicity caused by Aβ_1–42_ aggregation. **p < 0.01 ***p < 0.001 compared with the Aβ group.

We comprehensively evaluated the results of the previous peptide self-toxicity and ThT fluorescence intensity, and finally selected the CCK-8 method to evaluate the effect of the P21 peptide on the PC12 cytotoxicity induced by Aβ_1–42_, as shown in Figure 5. It can be seen from the figure that the inducer containing only Aβ_1–42_ can cause about 20% of cell death. Incubate the P21 polypeptide with Aβ_1–42_. The P21 polypeptide has a good inhibitory effect on the PC12 cytotoxicity caused by the aggregation of Aβ_1–42_ at a low concentration ratio (1:0.25 and 1:0.5). After the concentration ratio of P21 reached 1:1, with the increase of P21 concentration (1:2, 1:4), cell viability continued to increase, reaching the maximum at 1:4, which was close to the normal level, indicating that P21 can inhibit the aggregation process of Aβ_1–42_ Toxicity to PC12 cells.

### 3.7. Analysis of the binding force of peptides and Aβ_1–42_

**Figure 6:**
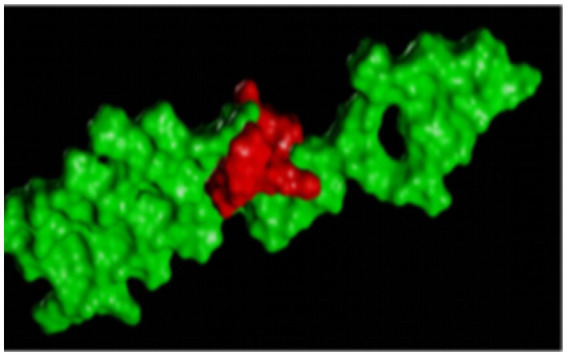
The three-dimensional structure diagram of the docking of the polypeptide and Aβ_1–42_, the red is the P21 in red, and the Aβ_1–42_ is in green.

**Figure 7:**
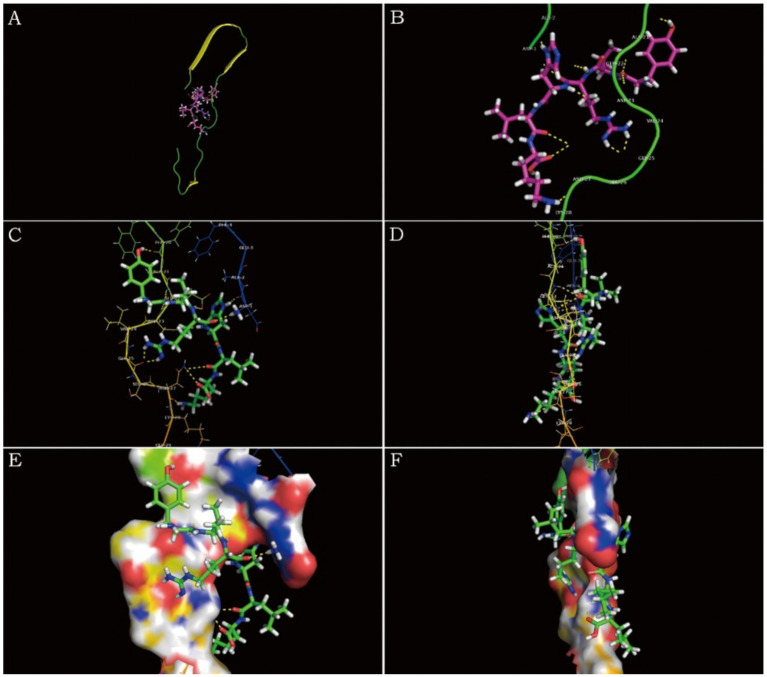
Schematic diagram of P21 and Aβ_1–42_ protein binding. **(A)** The three-dimensional structure diagram of the combination of P21 and Aβ_1–42_ is displayed as a whole. **(B–F)** Show the combination of P21 and Aβ_1–42_ from different perspectives.

Figure 6 is a diagram showing the relative position of P21 and Aβ_1–42_ protein after docking. P21 is represented in green, and Aβ_1–42_ is represented in red. The three-dimensional structure diagram of the combination of P21 and Aβ_1–42_ is shown in Figure 7. It can be seen from the figure that P21 is located in the center of the groove-like active pocket on the Aβ_1–42_ protein, which is in line with the Asp-1, Ala-21, Glu-22, Asp-23, Val-24 of the Aβ_1–42_ protein. The amino acids at Gly-25, Ser-26, Asn-27, and Lys-28 are connected by hydrogen bonds. The core hydrophobic region is composed of Ala-21 and Glu-22, which play a key role in the aggregation of Aβ_1–42_. It indicates the possible mechanism of action of the peptide we designed and verified the specific binding of the peptide and Aβ_1–42_ protein.

## 4. Discussion

In this study, the virtual peptide P21 (YVRHLKYVRHLK) designed by molecular docking has a good inhibitory effect on the protein aggregation of Aβ_1–42_ and reduces the neurotoxicity induced by Aβ_1–42_, which is consistent with the results of previous studies (Mondal et al., 2019; Kapadia et al., 2021). The mechanism of action may be that it has specific affinity for the hydrophobic amino acid residues in Aβ_1–42_ (Ala-21 and Glu-22, Aβ_21–22_), and inhibits the formation of β-sheets within Aβ_1–42_ and the relationship between β-sheets. The binding between them reduces the formation of soluble oligomers.

Abnormally precipitated Aβ in amyloid plaques becomes one of the hallmark proteins of brain nerve damage in AD patients, and promotes the deterioration of AD. The accumulation of Aβ protein is caused by the interaction between the hydrophobic center and the C-terminal molecule (Kapadia et al., 2021). At present, the development of anti-Aβ aggregation compounds is a promising approach in the treatment of AD, and many clinical trials are ongoing. A molecular dynamics study by Hong Zhou et al. using the core fragment of Aβ (KLVFFA) found that lanosterol binds to the Aβ residues Phe-19 and Phe-20 to form a hydrophobic site, which further induces the decomposition of Aβ protein and the β-sheet layer. Separate. The ThT fluorescence intensity in the cell experiment also showed that lanosterol can reduce the Aβ-induced cytotoxicity in PC12 cells and showed a good inhibitory effect (Zhou et al., 2019). In addition, a study showed that after vitamin K2 intervention, the number of apoptosis caused by Aβ accumulation was significantly reduced, and the ratio of Bax/Bcl-2 decreased, which had a protective effect on the cytotoxicity caused by Aβ deposition. Which has anti-apoptotic and antioxidant effects, may inactivate p38 The MAP kinase pathway is a valuable candidate protective substance against the progress of AD (Hadipour et al., 2020). Pradeepkiran et al. constructed a BACE1 pharmacophore with pepstatin, and screened it by molecular docking studies. It was found that there was an interaction between ligand 1 and BACE1, which reduced the activity of BACE1 and the levels of Aβ40 and 42. In addition, in mutant APP cells treated with ligand 1, mitochondrial biogenesis, mitochondrial fusion and synaptic activity increased, while mitochondrial fission decreased (Pradeepkiran et al., 2020).

In recent years, through the emergence of peptides that inhibit the aggregation of Aβ, extensive research has been conducted on peptides for the treatment of Alzheimer’s disease. However, these peptides have high specificity and low toxicity, which will make them candidate drugs for peptide inhibitors against Aβ aggregation (Fosgerau and Hoffmann, 2015). Peptides designed by computer simulation and applied to Alzheimer’s disease are becoming more and more popular and welcomed by researchers. At present, the peptide inhibitors of Aβ aggregation include LVFF, KLVFF, KLVFF modified peptide (QKLVFF), halogenated KLVFF peptide, FVFLM, and LF (Takahashi and Mihara, 2008; Kouza and Banerji, 2017; Khalili Samani and Mofid, 2020). The amyloid β-sheet mimics designed by Pin-Nan Cheng et al., KLVFFAE (Aβ_16–22_) and LFFFAED (Aβ_17–23_) peptides, have the effect of antagonizing Aβ protein aggregation and reducing Aβ toxicity (Cheng et al., 2012). Another study showed that KLVFF analog (QKLVFF, Aβ_15–20_) is a modified peptide inhibitor of Aβ protein aggregation, which can specifically bind to Aβ_1–40_ peptide, thereby inhibiting the formation of Aβ fibers (Findeis et al., 1999). Studies have shown that 10 μM iodinated KLVFF peptides (H_2_N-KLVFF (4-I) -CONH_2_) and 10 μM Aβ at a concentration ratio of 1:1 show the best resistance compared to other halogenated peptides. Aggregation activity (Khalili Samani and Mofid, 2020). Aggregation activity. The LF peptide (sequence Ac-KQKLLLFLEE-NH_2_) constructed using Aβ binding elements can form amyloid fibrils, which can effectively co-assemble with mature Aβ_1–42_ fibrils and effectively inhibit Aβ_1–42_ oligomerization (Takahashi and Mihara, 2008).In addition, the pituitary adenylate cyclase activating polypeptide (PACAP) attenuates Aβ-induced cell death in PC12 cells by increasing cAMP, and the inactivation of apoptotic factor (caspase-3) indicates that PACAP has a neuroprotective effect (Onoue et al., 2002).

The peptides synthesized by targeting Aβ aggregation have not only been extensively studied in *in vitro* experiments, but also in many *in vivo* experiments. For example, the tetrapeptide Ser-Leu-Lys-Pro (SLKP) studied by Pradhan K et al. showed significant neuroprotection against Aβ-mediated toxicity, promoted significant neurite growth, and maintained the growth of rat primary cortical neurons. Healthy form and cross the blood–brain barrier (BBB) (Pradhan et al., 2019). In this study, there is no *in vivo* study, which will be our follow-up work.

## 5. Conclusion

In summary, the aggregation of Aβ_1–42_ can cause the neurotoxicity of PC12 cells. The CScore of YVRHLKYVRHLK peptide molecule docking in the four peptides involved in this study (YVRHHHYVRHHH, YVRHSVYVRHSV, YVRHDLYVRHDL, YVRHLKYVRHLK) is 10.0608, the predicted activity score is 0.20 and the KD value is 5.385 × 10^−5^. The ThT and CCK-8 kit method found that the peptide itself is less toxic to PC12 cells at a concentration of 50 μM, and it has a significant inhibitory effect on the formation of Aβ_1–42_ aggregates when incubated with Aβ_1–42_ at a ratio of 1:1. It can significantly reduce the PC12 cytotoxicity induced by Aβ_1–42_, which further shows that the polypeptide YVRHLKYVRHLK designed in this study has neuroprotective effects, which may be due to the high degree of binding between the polypeptide and the amino acid residues 21–22 in Aβ_1–42_. Caused by the hydrophobicity. YVRHLKYVRHLK can be considered as a potential drug to prevent the progression of Alzheimer’s disease or as a pretreatment to slow down the progression of the disease.

## Data availability statement

The original contributions presented in the study are included in the article/supplementary material, further inquiries can be directed to the corresponding author.

## Ethics statement

The animal experimental protocol was approved by Scientific Ethics Committee of Henan University of Animal Husbandry and Economy (Zhengzhou, China).

## Author contributions

YW, SG, and JK designed the whole experiment, provided technical support and technical assistance. YW and SG conducted the experiments. SG and KW wrote the manuscript and analyzed data. KW and JK revised the manuscript. All the authors read and approved the manuscript.

## Funding

This research was supported by the National Natural Science Foundation of China (NNSFC), by grant NNSFC -31201878, U1204804, and U1604183. It has also been funded by the Excellent Youth Project of the Natural Science Foundation of Henan Province 202300410193.

## Conflict of interest

The authors declare that the research was conducted in the absence of any commercial or financial relationships that could be construed as a potential conflict of interest.

## Publisher’s note

All claims expressed in this article are solely those of the authors and do not necessarily represent those of their affiliated organizations, or those of the publisher, the editors and the reviewers. Any product that may be evaluated in this article, or claim that may be made by its manufacturer, is not guaranteed or endorsed by the publisher.

## Footnotes

^1^ http://distilldeep.ucd.ie/PeptideRanker/

^2^ http://www.peptide-ligand.cn/tools/

^3^ http://crdd.osdd.net/raghava//toxinpred/
